# *Petrocosmeaduyunensis* (Gesneriaceae), a new species from Guizhou, China

**DOI:** 10.3897/phytokeys.181.68635

**Published:** 2021-08-16

**Authors:** Sheng-Hu Tang, Jia-Wen Yang*, Cong-Rui Li, Qing Zhou

**Affiliations:** 1 Guizhou Botanical Garden, CN-550000 Guiyang, China; 2 Guizhou Academy of Forestry, CN-550000 Guiyang, China; 3 Gesneriad Conservation Center of China (Guizhou), Guizhou Botanical Garden, CN-550000 Guiyang, China

**Keywords:** Didymocarpoideae, flora of China, morphology, taxonomy

## Abstract

Plants belonging to the genus *Petrocosmea* are rare and small herbs difficult to find in the wild. In the present study, a new species, *Petrocosmeaduyunensis*, from Guizhou, China, is described. The species is most similar to *P.leiandra* and differs from it by a distinctly recurved abaxial corolla lip, free anthers and included pistil. Detailed morphological comparisons are given. One population with about 100 mature individuals was found at the type locality. This new taxon was assessed as “Data Deficient” (DD) according to the IUCN standards.

## Introduction

The genus *Petrocosmea* Oliv. belongs to the family Gesneriaceae, subfamily Didymocarpoideae (Weber et al. 2013). According to an earlier revision of the genus by Wang (1985), 27 species and four varieties were recognised and classified into three sections, *viz.*, sect. Anisochilus Hemsl., sect. Deinanthera W.T.Wang, and sect. Petrocosmea. In 2015, the first phylogenetic analyses of the genus were presented, 33 species and three varieties were classified into five sections, *viz.*, sect. PetrocosmeaOliv.,sect.Anisochilus Hemsl., sect. Minor Zhi J. Qiu, sect. Barbata Zhi J. Qiu, and sect. Deinanthera W.T. Wang (Qiu and Liu 2015; Qiu et al. 2015). Presently, the genus comprises 56 species with three varieties (GRC 2021). Its native range is from the eastern Himalayas to central China and Indo-China (POWO 2021). The distribution of most of *Petrocosmea* species is restricted to relatively small geographical areas (Wang et al. 1998; Wei and Wen 2009). South and southwestern China are the centres of gesneriad diversity (Xu et al. 2017). Guizhou is a province that lies in the southwest of China. To date, 14 taxa of *Petrocosmea* have been recorded in Guizhou, and the type localities of eight taxa were in Guizhou (Wang 1984; Gou et al. 2010; Han et al. 2017, 2019).

In November 2020, during field work in Duyun county, Guizhou province, we collected some living plants belonging to the genus *Petrocosmea*. In April 2021, when the plants flowered in the greenhouse of the Guizhou Botanical Garden, they had distinctly recurved abaxial corolla lips and included pistil. In the same month, we visited the type locality again and collected flowering specimens. The plants were classified as belonging to sect. Minor, which mostly possesses a highly fused upper lip and forms a carinate-plicate shape on the upper lip. They were most similar to *P.leiandra* (W.T.Wang) Z. J. Qiu in the shape of leaf blades and corolla limb, and anther dehiscence. After thorough comparisons, we concluded that the plants represented a new species.

## Methods

Morphological observations of flowering plants were performed in the field and in the greenhouse. All morphological characteristics were observed under a dissecting microscope (Olympus SZ61, Tokyo, Japan), and descriptions were made following the terminology used in Wang et al. (1998). The relevant literature was consulted, including Xu et al. (2011), Qiu et al. (2012), Zhang et al. (2013), Han et al. (2018), Wen (2019), Jiang et al. (2020), Qiu et al. (2020), Huang and Xin (2021), in addition to those mentioned in the introduction. The images of type specimens available in virtual herbaria and databases, including RBGE (https://data.rbge.org.uk/search/herbarium/), Kew Herbarium Catalogue (http://apps.kew.org/herbcat/navigator.do), MNHN (https://www.mnhn.fr/en), PE (https://pe.ibcas.ac.cn/index.html), and iPlant (http://www.iplant.cn/) were also examined. In order to obtain comparable morphological data for living plants, flowering plants belonging to the new taxon and similar species were collected from their type localities. The type specimens of the new taxon were collected by Sheng-Hu Tang in the field.

## Taxonomic treatment

### Petrocosmea duyunensis

Taxon classificationPlantaeLamialesGesneriaceae

Sheng H.Tang sp. nov.

6C58FE78-1CED-5FA6-8F64-56334660EFC8

urn:lsid:ipni.org:names:77219124-1

Figure 1

#### Type.

China Guizhou Province, Duyun County, Doupeng Mountain, 26.37 N, 107.37 E, about 1047 m a.s.l., 23 April 2021, *Sheng-hu Tang 2021001* (Holotype: IBK!; Isotype: CSH!)

#### Diagnosis.

*Petrocosmeaduyunensis* is most similar to *P.leiandra* in the shape of leaf blades and corolla limb, and in the indumentum of peduncles, calyx, and ovary. However, it differs from the latter in the following characteristics: apex of abaxial lip lobes of corolla acute and reflexed (*vs.* round and not reflexed), filaments densely glandular puberulent (*vs.* glabrous or sparsely pilose), pistil 4.7–5.8 mm long, included (*vs.* ca. 9 mm long, exserted), ovary ovoid (*vs.* narrowly ovoid), style distinctly curved, sparsely glandular puberulent, and 2.7–3.7 mm long (*vs.* slightly curved, glabrous, and ca. 7 mm long).

**Figure 1.**
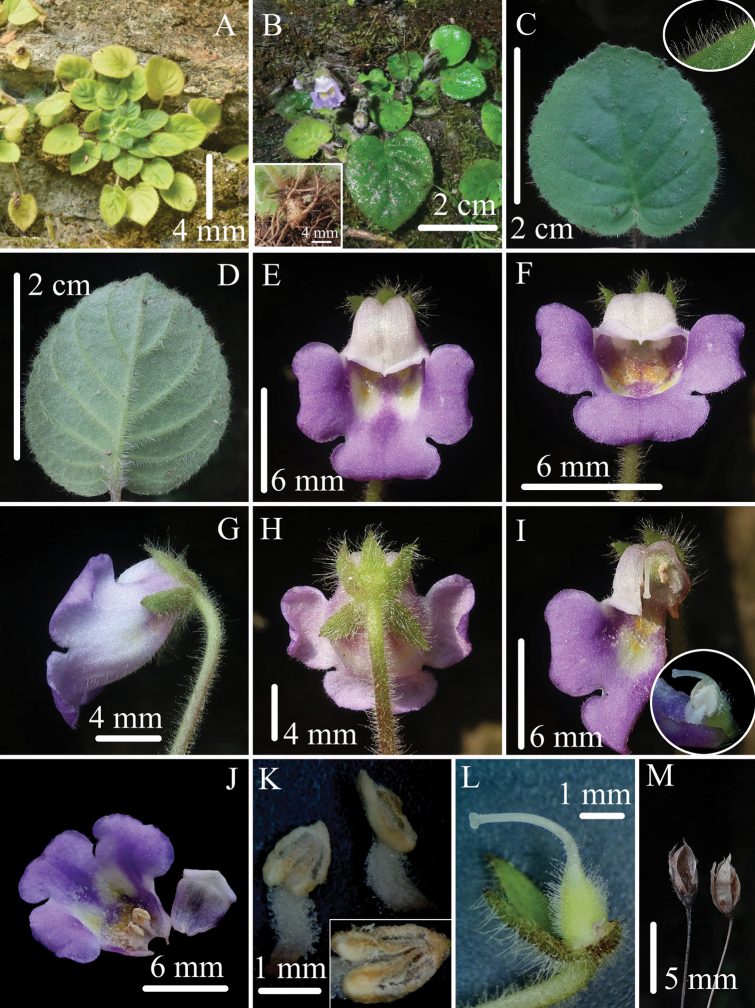
*Petrocosmeaduyunensis* Sheng H.Tang sp. nov. **A** habitat **B** flowering plant, rhizome and fibrous roots (inset) **C** adaxial surface of leaf blade and indumentum (inset) **D** abaxial surface of leaf blade **E, F** flowers in front view **G** flower in side view **H** flower in back view **I, J** dissected corolla, stamens and pistil with corolla removed (inset) **K** stamens and anther (inset) **L** pistil with abaxial calyx lobes, and adaxial lobes removed **M** capsules of previous year. (Photographed by Sheng-Hu Tang).

#### Description

. Perennial herbs; rhizomes short, 7–8 mm in length. Leaves 10–30, basal, crowded, with long petioles up to 5 cm, petioles densely glandular pilose and sparsely villous; leaf blades papery when dry, their outer blades ovate or suborbicular, 1.6–2.8 × 1.6–2.6 cm, the blade apex obtuse or rounded, base cordate, sometimes oblique, margin crenate, adaxial blade surface densely glandular pilose, abaxial blade surface densely pilose and sparsely glandular pilose, lateral veins adaxially impressed, abaxially conspicuous, 4–6 on either side of the midrib. Cymes 2–7, 1–3 flowers per cyme; peduncles 1.8–4.0 cm long, densely glandular pilose and sparsely villous; bracts 2, opposite, linear, 2–2.2 × 0.7–0.8 mm, sparsely puberulent outside, glabrous inside. Pedicels 1.0–2.5 cm long, densely glandular pilose and sparsely villous. Calyx zygomorphic, 5 lobes apex acuminate, densely pilose and sparsely glandular pilose outside, glabrous inside; adaxial calyx lib 3.9–4.2 mm in length, 3-lobed to below the middle, lobes narrowly triangular, 2.2–2.6 × 1.0–1.4 mm; abaxial calyx lib 2-lobed near the base, lobes oblong-triangular, 3.9–4.3 × 1.4–1.5 mm. Corolla blue-purple, 10.7–12.5 mm, glandular pubescent outside, glabrous inside; tube 4.6–5.4 mm, two yellow spots inside the tube beneath the anthers, throat violet, two white spots in the throat; adaxial corolla lip distinctly short, 1.0–2.0 mm in length, indistinctly 2-lobed, lobes 0.5–1.2 × 2.6–2.8 mm, reflexed slightly; abaxial corolla lip 6.8–8.1 mm, 3-lobed, lobes 2.6–3.0 × 4.6–7.1 mm, broadly ovate, with acute and reflexed apex. Stamens 2, free, adnate to the corolla tube at the base, included; filaments 1.5–2.0 mm in length, 0.5–0.7 mm in diameter, densely glandular puberulent, slightly curved near the middle; anthers ovate, 1.5–1.6 × 1.0–1.1 mm, dorsifixed; thecae parallel, dehiscing longitudinally. Staminodes 3, inconspicuous, adnate to the corolla tube at the base, glabrous. Pistil 4.7–5.8 mm long, included; ovary densely glandular pilose, ovoid, ca. 2.0 mm long, 1.2–1.6 mm in diameter; style 2.7–3.7 mm long, 0.2 mm in diameter, sparsely glandular puberulent below the middle, curved above the base at an angle approaching 90°; stigma capitate, 0.3 mm in diameter. Capsule 4.8–6.2 mm long, 1.9–2.0 mm in diameter, ovoid, dehiscing loculicidally to base, valves 2.

#### Phenology.

Flowering from April to May, fruiting in the wild is unknown, only capsules of the previous year were observed.

#### Etymology.

The new taxon is named after the type locality, Duyun county, China.

#### Vernacular name.

The Chinese name is “Dū Yún Shí Hú Dié” (都匀石蝴蝶).

#### Distribution and habitat.

To date, only a single population has been found in Doupeng mountain, Duyun county, Guizhou province, China. The plants were found growing on a moist shady cliff in a valley at an altitude of ca. 1047 m.a.s.l. The main companion species were *Sloaneahemsleyana* (Ito) Rehd. et Wils., *Corylopsismultiflora* Hance, *Pittosporumglabratum* Lindl., and *Oreochariselegantissima* (H.Lév. & Vaniot) Mich.Möller & W.H. Chen.

#### Conservation status and IUCN Red List category.

One population with about 100 mature individuals was found at the type locality. The habitat is in a nature reserve protected by the local government. It is highly likely that more populations are present in the area. Before further investigations, this species should be assessed as “Data Deficient” (DD) according to the IUCN standards (IUCN 2019).

#### Notes.

**Figure 2.**
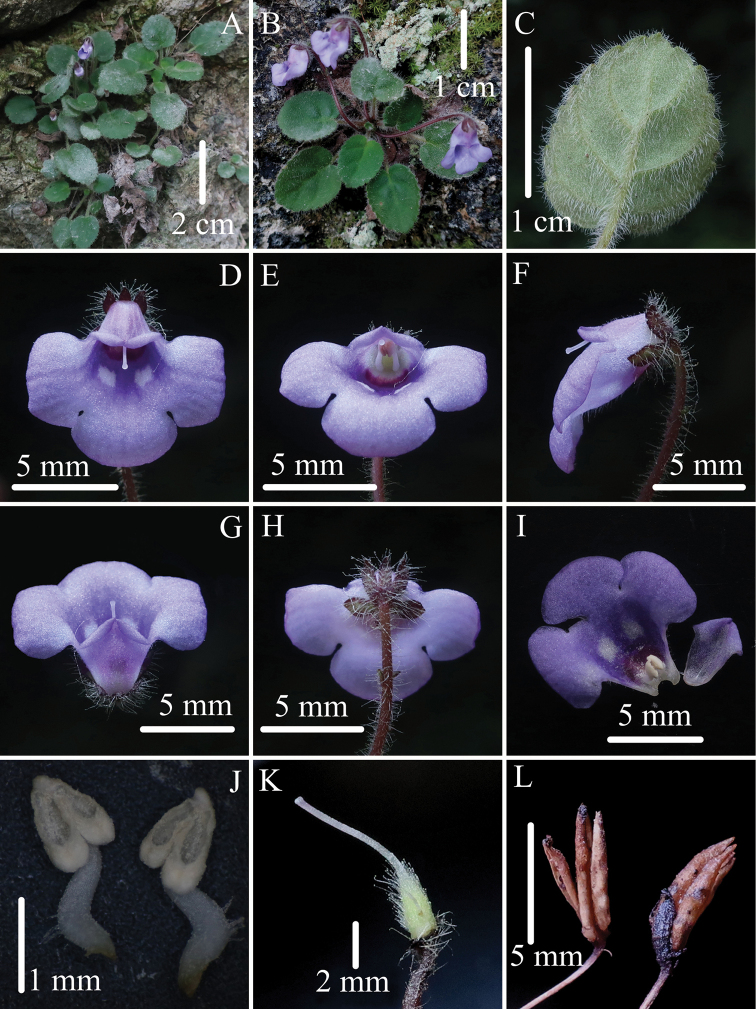
*Petrocosmealeiandra* (W.T.Wang) Z. J. Qiu **A** habitat **B** flowering plant **C** abaxial surface of leaf blade **D, E** flower in front view **F** flower in side view **G** flower in top view **H** flower in back view **I** dissected corolla **J** stamens **K** pistil with calyx removed **L** capsules of previous year. (Photographed by Sheng-Hu Tang).

Although *Petrocosmeaduyunensis* has a short adaxial corolla lip, the pistil is still included in the corolla. This is because the style is relatively short and distinctly curved. The plant possesses two free anthers. These characteristics differ from those of other *Petrocosmea* species. Morphologically, the species is similar to *P.leiandra* (Fig. 2), and detailed morphological comparisons are shown in Table 1.

**Table 1.** Detailed comparison between *Petrocosmeaduyunensis* and *P.leiandra*.

Character / species	* P. duyunensis *	* P. leiandra *
Apex of abaxial corolla lip lobes	acute and reflexed	round and not reflexed
Stamen filament indumentum	densely glandular puberulent	glabrous or sparsely pilose
Anthers	free	coherent at apex
Pistil length	4.7–5.8 mm	ca. 9 mm
Pistil position	included in the corolla	exserted from the corolla
Ovary shape	ovoid	narrowly ovoid
Style indumentum	sparsely glandular puberulent	glabrous
Style length	2.7–3.7 mm	ca. 7 mm
Style shape	with a bent approaching 90°	slightly curved

## Supplementary Material

XML Treatment for Petrocosmea duyunensis
